# The effects of necrotic lesion size and orientation of the femoral component on stress alterations in the proximal femur in hip resurfacing - a finite element simulation

**DOI:** 10.1186/1471-2474-15-262

**Published:** 2014-08-05

**Authors:** Ching-Lung Tai, Yung-Chou Chen, Pang-Hsin Hsieh

**Affiliations:** 1 Graduate Institute of Medical Mechatronics, Department of Mechanical Engineering, Chang Gung University, Taoyuan, Taiwan; 2 Department of Orthopaedic Surgery, Chang Gung Memorial Hospital, Chang Gung University College of Medicine, Taoyuan, Taiwan

**Keywords:** Hip resurfacing, Femoral head necrosis, Stress shielding, Finite element analysis

## Abstract

**Background:**

Due to the advantages of its bone-conserving nature, hip resurface arthroplasty (HRA) has recently gained the interest of orthopedic surgeons for the treatment of young and active patients who have osteonerosis of the femoral head. However, in long-term follow-up studies after HRA, narrowing of the femoral neck has often been found, which may lead to fracture. This phenomenon has been attributed to the stress alteration (stress shielding). Studies addressing the effects of necrotic size and the orientation of the implant on stress alterations are lacking.

**Methods:**

Computed tomography images of a standard composite femur were used to create a three-dimensional finite-element (FE) intact femur model. Based on the intact model, FE models simulating four different levels of necrotic regions (0°, 60°, 100°, 115°) and three different implant insertion angles (varus 10°, neutral, valgus 10°) were created. The von Mises stress distributions and the displacement of the stem tip of each model were analyzed and compared for loading conditions that simulated a single-legged stance.

**Results:**

Stress shielding occurred at the femoral neck after HRA. More severe stress shielding and an increased displacement of the stem tip were found for femoral heads that had a wider necrotic lesion. From a biomechanics perspective, the results were consistent with clinical evidence of femoral neck narrowing after HRA. In addition, a varus orientation of the implant resulted in a larger displacement of the stem tip, which could lead to an increased risk of implant loosening.

**Conclusions:**

A femoral head with a wide necrotic lesion combined with a varus orientation of the prosthesis increases the risk of femoral neck narrowing and implant loosening following HRA.

## Background

Osteonecrosis of the femoral head (ONFH) is a devastating disease that typically affects patients who are in the third through fifth decades of life. The development of ONFH is usually progressive, and it often leads to the collapse of the femoral head and the subsequent destruction of the hip, requiring the joint to be reconstructed using a prosthetic replacement.

Hip resurface arthroplasty (HRA) has several advantages compared with traditional total hip arthroplasty (THA), including a reduced rate of dislocation due to the larger diameter of femoral head, reduced wear of metal-on-metal interface, a larger range of motion and its bone-conserving nature [1-3]. Consequently, HRA is considered suitable for the treatment of young and active patients who have ONFH. Although promising results have been reported in some short-term follow-up studies [4-6], aseptic loosening and narrowing of the femoral neck, which may lead to femoral neck fractures have also been reported in long-term follow-up studies [7-10].

In the surgical procedure for HRA, the necrotic bone tissue is removed and replaced with cement. There are some concerns regarding this procedure. Previous studies [11,12] have reported that necrotic bone replaced with cement can induce alterations in femoral strain. To the best of our knowledge, however, literatures addressing the combined effects of necrotic lesion size and orientation of the implant on postoperative mechanical performance, including altering the stress distribution and the risk of implant loosening, are lacking. Therefore, this study was designed to examine how the necrotic lesion size and implant orientation affect the mechanical behavior of the operated femur. We hope to achieve a thorough understanding of femoral neck narrowing and aseptic implant loosening following HRA.

## Methods

### Generation of the 3-D solid model

**Figure 1:**
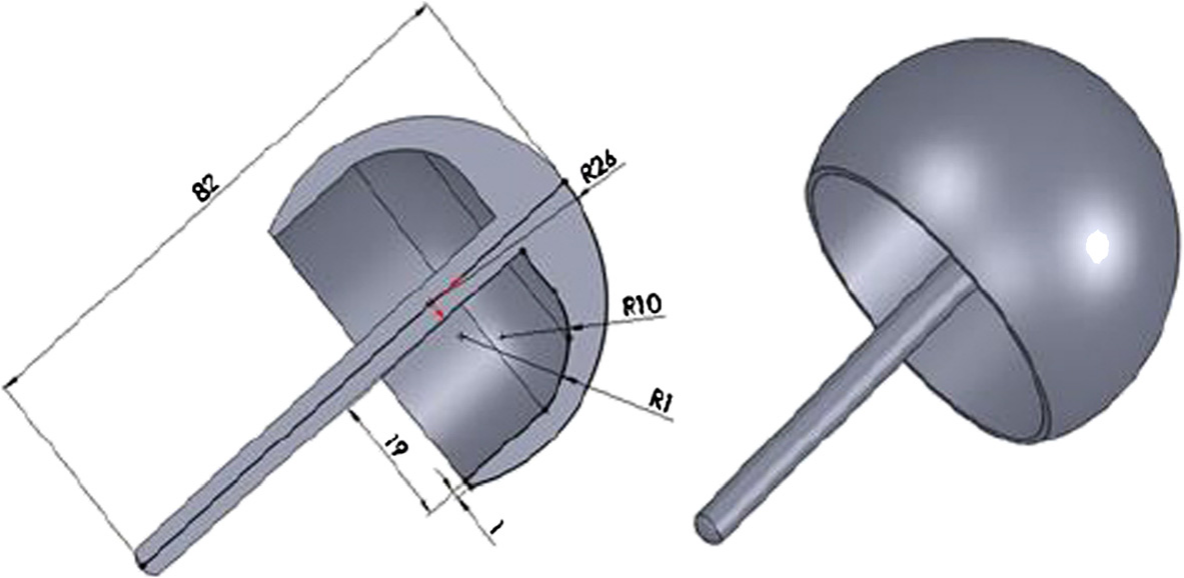
Three-dimensional solid model of the femoral component for HRA.

A commercially available synthetic femur (Model: #3306, Pacific Research Laboratory Inc., Vashon Island, WA, USA) was used as the study model for HRA. 3-D Solid models of a standard composite femur were created using computed tomography (CT) images. CT images of the intact femur were obtained at 1.25 mm intervals in the transverse planes starting from the femoral head, using a GE Hi-speed scanner (General Electric, Milwaukee, WI, USA). The resolution for each CT image was 512 by 512 pixels, the field of view was 320 mm, and the pixel size was 0.625 mm/pixel. The obtained cross-sectional images of the femur were transferred to an automatic contouring program to detect the contours between the cortical and cancellous bone. The parallel stacked contours were then input into the Solidworks CAD software (SolidWorks 2004, SolidWorks Corp. Boston, MA, USA) to reconstruct a 3-D solid model of the intact femur. The solid model of the implant was generated based on the measurement of a commercially available product (Durom hip resurfacing, Zimmer, Warsaw, IN, USA) (Figure 1).

### Definition of necrotic lesion size and alignment of femoral component

**Figure 2:**
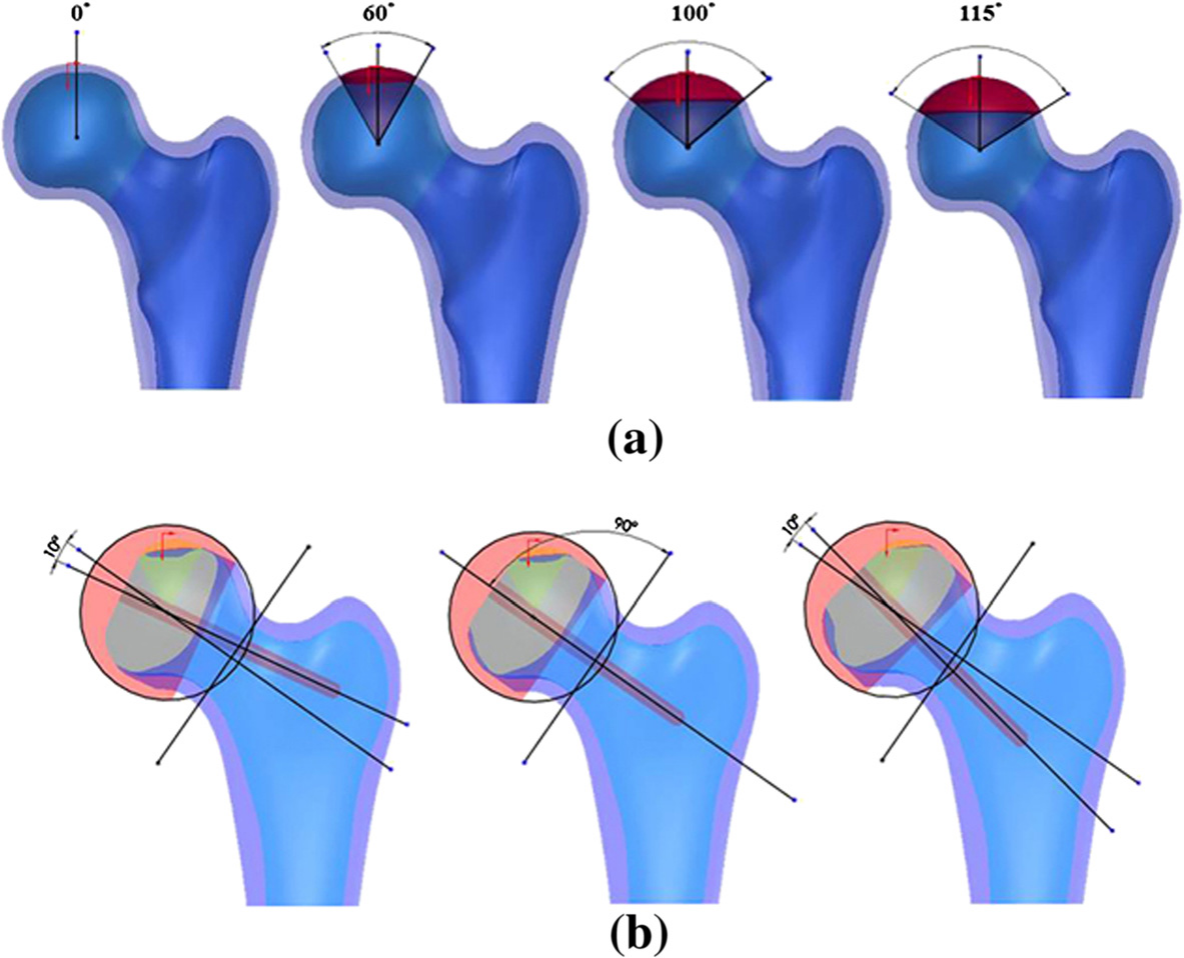
**Designation of solid models for specific necrotic lesion size and implant orientation. (a)** Femora with 0°, 60°, 100° and 115° necrotic lesion size (from left to right), **(b)** Femora with implants aligned varus 10°, neutral and valgus 10° (from left to right).

Twelve models simulating four different sizes of necrotic lesion (0°, 60°, 100°, 115°) and three different alignments of the femoral component (10° varus, neutral, 10° valgus) were created by modifying the intact model (Figure 2). The necrotic lesion was arbitrarily defined as a conoid projecting from the center of the femoral head, and the axis of the conoid (necrotic axis) was aligned with the line that connected the most superior point of the femoral head and the center of the femoral head. Four different sizes of conoids with cone angles of 0°, 60°, 100° and 115° were chosen to represent the intact, low, intermediate and high risk groups, respectively [13]. Models with specific necrotic lesion size but different alignments of the femoral component for HRA were devised by positioning the stem of the femoral component at different angles with respect to the neck axis. Three models were created for each specific lesion size: one resurfaced with the arthroplasty oriented in line with the femoral neck (neutral), one with the implant positioned at a varus orientation of 10° less than the neutral axis line and one with the implant positioned at a valgus orientation of 10° greater than the neutral axis line. The assumptions of bone/cement, cement/implant and stem/bone interfaces were adopted from the literature [14]. The inner surface of the implant and the necrotic lesion region were modeled as being cemented. Both the bone/cement and cement/implant interfaces were assumed to be fully bonded. The stem was modeled as debonded, simulating no contact between the stem and bone. Full details of the assignments of the bone/cement, cement/implant and stem/bone interfaces are presented by Taylor [14].

### Loading and boundary conditions

**Figure 3:**
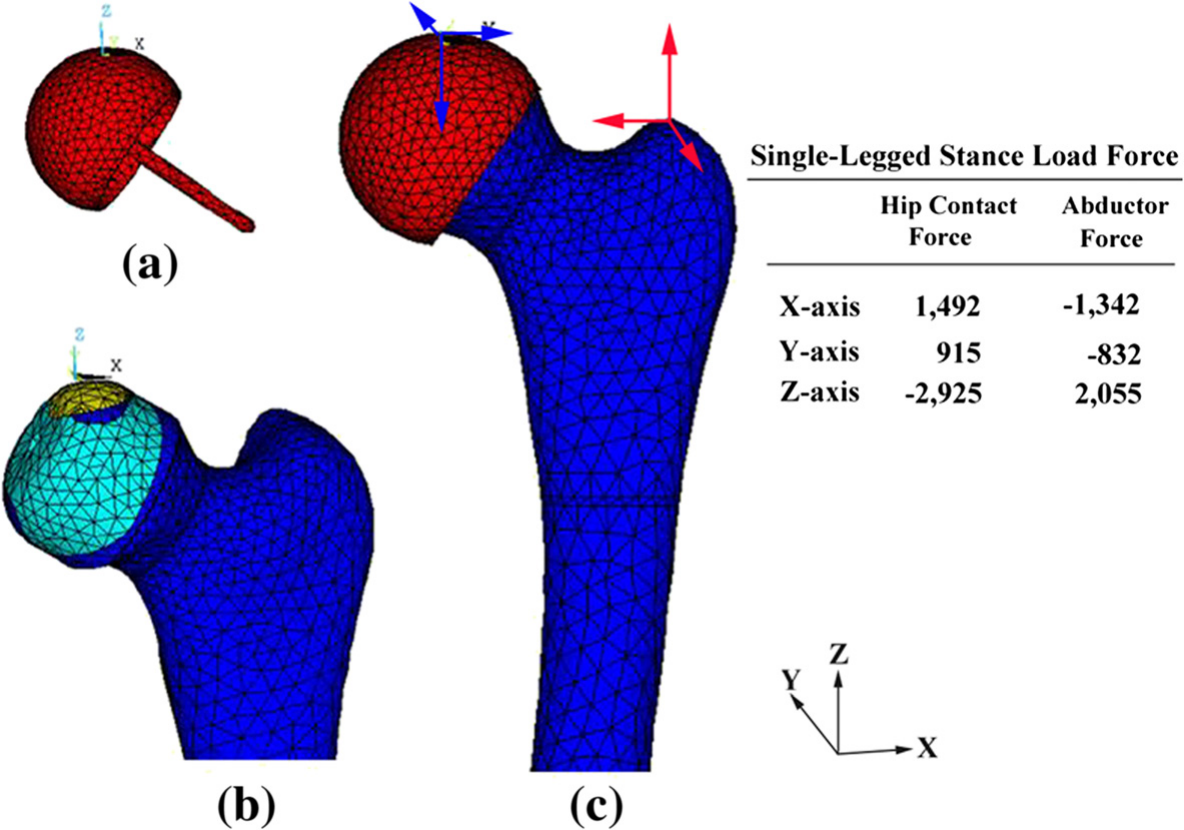
**Tetrahedral element models and applied loading. (a)** Femoral component, **(b)** Femur with necrois, and **(c)** Femur after HRA.

The element type used for all materials in the FEA model was a 10-node, isoparametric tetrahedral element. A loading condition simulating single-legged stance was adopted from the literature [15] and applied to all FE models (Figure 3). All material properties were modeled as a homogeneous linear elastic continuum exhibiting isotropic properties. The Poisson’s ratios used for cortical bone, cancellous bone, the implant and the bone cement were 0.3, 0.22, 0.3 and 0.19, respectively, and the moduli of elasticity were 15.1 GPa, 445 MPa, 200 GPa and 2000 MPa, respectively [16]. The FE analysis for each of the 12 models was performed using a commercial finite-element package (Ansys 12.0, Ansys, Inc, Canonsburg, PA, USA). The von Mises stress distributions and the displacement of stem tip of each model were analyzed and compared.

### Validation of FEM model

For FEA validation, an experimental test of an intact synthetic femur was conducted on a MTS materials testing machine (Bionix 858, MTS Corporation, Minneapolis, MN, USA). A linear variable differential transformer (LVDT, DT-10 F, Kyowa, Tokyo, Japan) was attached vertically on the inferior aspect of the femoral head. The vertical displacement of the femoral head of the intact femur was measured under a 2000 N compressive load on the femoral head. The vertical displacement at the inferior aspect of the femoral head was also obtained from an intact FEA model that was subjected to an identical 2000 N compressive load. The experimental and analytical results were compared to validate the FEA model.

## Results

### The effect of necrotic lesion size on the stress distribution

**Figure 4:**
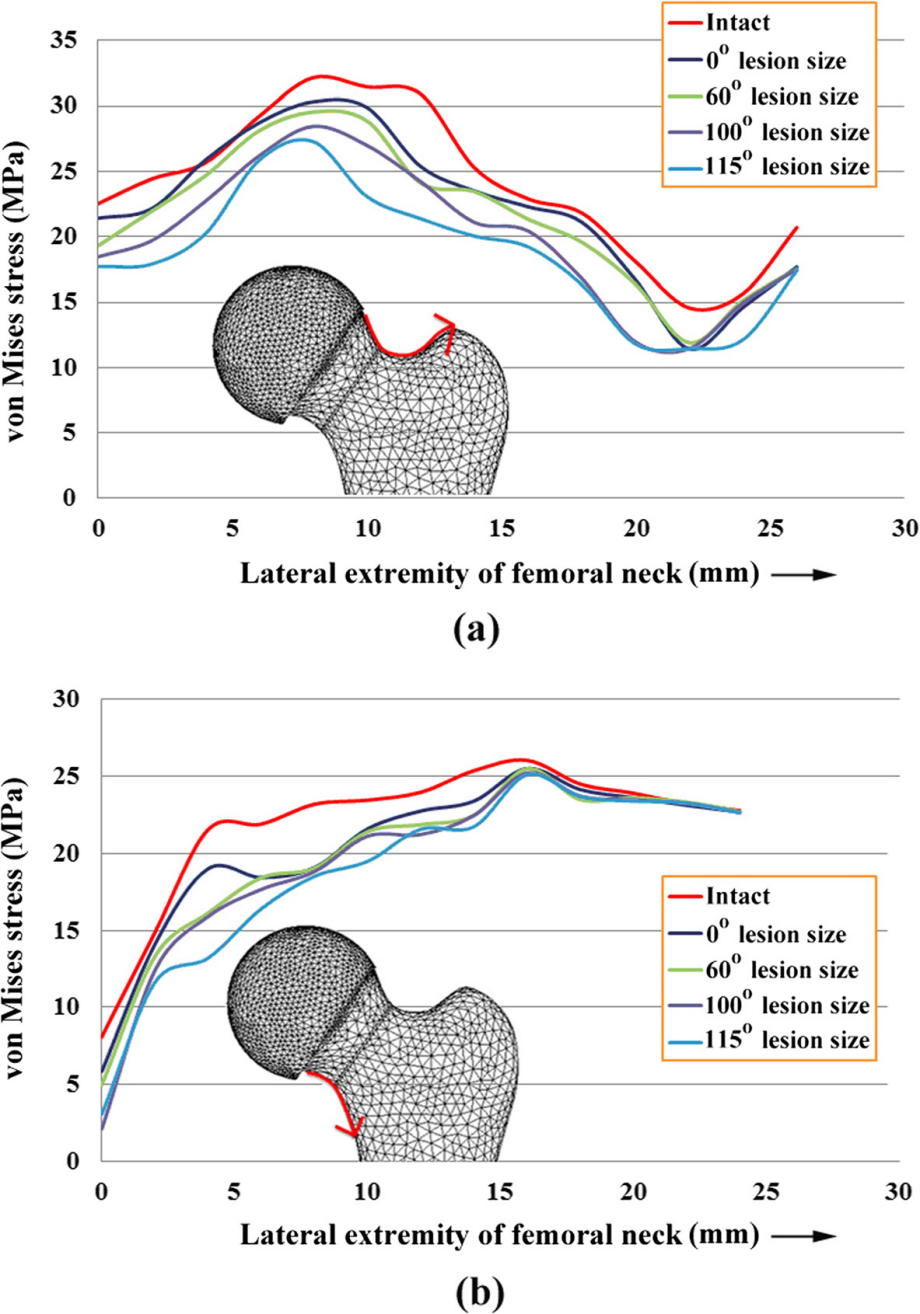
von Mises stress (a) on the superior region of the femoral neck and (b) on the inferior region of the femoral neck, intersected with the frontal plane, for an intact femur and femora with 0°, 60°, 100° and 115° necrotic lesion sizes and HRA with a neutral orientation.

The von Mises stress on the superior and inferior regions of the femoral neck intersected with the frontal plane for both the intact model and the models with various necrotic lesion sizes for HRA with a neutral orientation, as shown in Figure 4. The von Mises stress on the most superior point of the femoral neck for the intact femur and femora with 0°, 60°, 100° and 115° lesion sizes were 32.162 MPa, 30.323 MPa, 29.55 MPa, 28.445 MPa and 27.293 MPa, respectively. Thus, the intact femur exhibited the highest stress. Following HRA, however, the stress decreased with increasing lesion size. This result implies that more severe stress shielding occurred for femur with larger lesion sizes.

### The effect of implant orientation on the displacement of the stem tip

**Figure 5:**
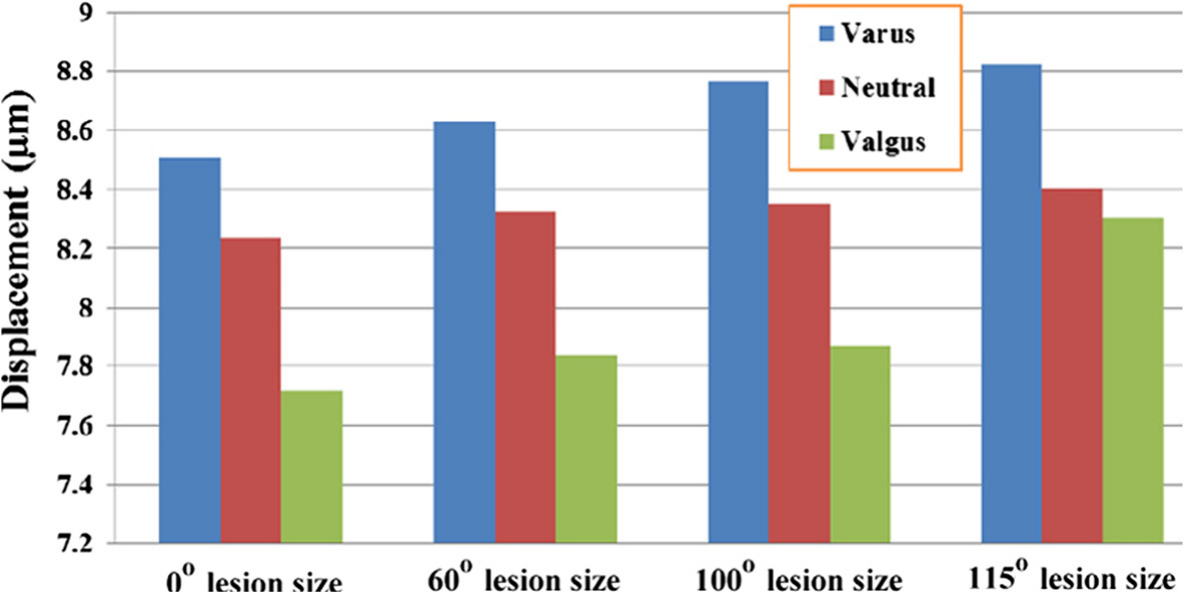
Displacement of the stem tip for femora with various necrotic lesion sizes resurfaced with varus, neutral and valgus implant alignment.

The displacement of the stem tip for femora with various necrotic lesion sizes resurfaced with 10° varus, neutral and 10° valgus aligned implants is shown in Figure 5. For femur with a 60° lesion size, the displacement of the stem tip for femoral components with varus, neutral and valgus orientations was 8.631 μm, 8.328 μm and 7.837 μm, respectively. For femur with a 100° lesion size, the stem tip displacements were 8.768 μm, 8.350 μm and 7.867 μm for the varus, neutral and valgus orientations, respectively. For femur with a 115° lesion size, the displacement of the stem tip for varus, neutral and valgus orientations was 8.825 μm, 8.405 μm and 8.303 μm, respectively. These results indicate that for femur with an identical lesion size, the varus implant orientation resulted in the highest stem tip displacement, whereas the valgus orientation resulted in the lowest displacement. Furthermore, regardless of the implant orientation (varus, neutral or valgus), the displacement of the stem tip tended to increase with increasing lesion size.

## Discussion

The finite element method has become a useful tool in analyzing the stresses in structures of complex shapes, loading and material behavior. Numerous applications in orthopedics have been presented, and the models developed have successfully predicted the mechanical characteristics of skeletal components in interesting circumstances. There are also ample precedents for the use of this method in studying femoral head osteonecrosis and hip resurfacing arthroplasty. Liu et al. [17] designed a three-dimensional finite element model to demonstrate stress changes in the necrotic femoral head with various lesion sizes. They concluded that femoral heads with larger necrotic lesions have a higher stress concentration and a higher risk of collapse. Tran et al. [18] simulated and analyzed a femur that was treated by core decompression with a bone substitute using a finite-element model. Their results demonstrated that the entrance point should be located on the proximal subtrochanteric region to reduce the risk of subtrochanteric fracture. As for previous studies that used FEM to evaluate the biomechanical behavior of the hip resurfacing arthroplasty, Radcliffe and Taylor [12] investigated the influence of the varus–valgus orientation on load transfer within the resurfaced proximal femur. They concluded that the valgus alignment of the resurfacing arthroplasty is preferential to the varus alignment because the former induces a more physiological strain pattern and reduces the risk of femoral neck fracture. Watanabe et al. [16] performed a finite element analysis using three-dimensional models to examine the biomechanical characteristics of the femoral component in HRA. Stress shielding was observed in the anterosuperior regions on the cancellous bone cross-sections near the cup rim. This stress alteration may lead to complications such as femoral neck fractures in patients with osteopenic bone and long-term loosening. Ong et al. [19] examined the effects of the fixation method and the interface conditions on the biomechanics of the femoral component of the Birmingham hip resurfacing arthroplasty using a three-dimensional computer model of the hip. Their results indicated that proximal femoral stresses and strains were non-physiological when the Birmingham hip resurfacing femoral component was fixed to bone. Bone resorption was predicted in the inferomedial and superolateral bone within the Birmingham hip resurfacing shell. Taylor [14] examined the influence of various metaphyseal stem configurations on load transfer within the femoral head. He concluded that increasing both the stem diameter and the percentage of the stem length in contact with bone increased the degree of strain shielding.

Although several of the reports mentioned above showed good results in predicting the mechanical behavior of the femoral head with a certain necrotic lesion, FEM studies considering changes in the stress distribution due to changes in the necrotic lesion size along with variations in the orientation of the femoral component in femoral resurfacing have not been reported. Previous studies comparing the mechanical performance of different extents of necrosis and stem orientation accompanied with femoral resurfacing are lacking. A study focused on the basic principles of femoral resurfacing is still of paramount importance to avoid unnecessary complications.

The validation and convergence of the FEM model must be taken into account before its interpretation. For model validation, the predicted displacement from FEA and the measured displacement from the experiment were 4.92 mm and 5.21 mm, respectively. The percentage error compared with the experimental result was small (5.57%). The convergence of the FEM models used in this study was justified by the total strain energy of the structure. Six models with different numbers of elements and nodes were created to perform the convergence test, and the results of the total strain energy for the six models were all within 5%. The model with the finest mesh was used in this study. The validity and convergence of the FEA model was thus demonstrated from the above procedures. The consistent results of the finite element and in vitro testing imply that the simulations are reliable.

HRAs are appealing procedures because the potential for bone conservation and the non-violation of the femoral shaft make it a less invasive option. Therefore, HRA may be suitable for young and highly active patients with ONFH. Numerous reports [20-22] support the use of HRA for ONFH, even when some necrotic bone remains, but the influence of the lesion size before resurfacing on the stability of the femoral components is unclear. Among the various types of HRA, the clinical outcome is very controversial because of the inconsistent results obtained by different authors [23-26]. The differences reported may be related to differences in the extent of necrosis, alignment of implant, and surgical techniques. The main reasons for failure include postoperative narrowing of the femoral neck [9,10] and loosening of the femoral head [23,24]. Fracture of the femoral neck may be attributed to femoral neck narrowing following HRA, whereas loosening of the femoral head has been attributed to the over-displacement of the implant stem.

The etiology of femoral neck narrowing after HRA is unknown. It may be the result of avascular circulation to the femoral head and neck, impingement, an inflammatory response or bone remodeling due to stress shielding. Based on Wolff’s law of bone remodeling, if the loading on a bone decreases, it will become less dense and weaker, which can change the shape of the bone because there is no stimulus for the continual remodeling that is required to maintain bone mass [27]. As observed in Figure 4, the magnitudes of von Mises stress on both the superior and inferior aspects of the femoral neck were smaller for the models with HRA compared with the intact model. A more severe reduction in the von Mises stress was found for femoral heads that had a larger necrotic lesion size. The stress shielding phenomenon could be attributed to the relatively high elasticity of the cement compared with the necrotic lesion being replaced. The result may indicate that altered loading of the femoral neck results in narrowing of the femoral neck. From a biomechanics perspective, our results provide a convincing explanation for the clinical outcome of neck narrowing after hip resurfacing arthroplasty [9,10]. Furthermore, we found that a wide necrotic lesion combined with a varus implant orientation caused the highest stem tip displacement (Figure 5), implying that a larger necrotic lesion with varus component placement is associated with implant loosening and implant failure. These findings are consistent with clinical experience, which has shown an increase rate of failure for varus implanted prostheses [12,28].

There are a number of limitations in the present study. First, the geometry and the linear, elastic, homogeneous material properties of a standard composite femur were used rather than the values for femurs from actual patients. One benefit of using a standard composite femur is that it eliminates the variations between subjects. However, the drawback is that this approach overlooks the effects of the nonlinear, inelastic, and non-homogeneous material properties of bone. Second, the only loading condition considered was the single-legged stance of gait. Further investigation on the effects of other loading conditions might be necessary in the future. Third, the interfaces on the implant/cement and cement/bone were assumed to be fully bonded, without considering the loosening of the implant. Therefore, the results from the FEA might only be interpreted for well-fixed conditions without implant loosening. Fourth, the necrotic lesion was arbitrarily designated to the most superior point of the femoral head. However, we demonstrated that better planning for femoral resurfacing could be achieved by predicting the stress distribution for different extents of necrosis. Last, the FE model was validated based on the intact condition without HRA, which may have an impact on the analytic results for the post-operative FE models. However, the boundary conditions including material properties, element types and element length are identical for FE models with or without HRA, and we believe that our results provide useful information to orthopedic surgeons performing HRA for patients with femoral head osteonecrosis.

## Conclusion

Necrotic lesion size and the orientation of the prosthesis have a considerable effect on the stress distribution in the femoral neck and on the postoperative stability of the implant after HRA. A femoral head with a wide necrotic lesion combined with a varus orientation of the prosthesis increases the risk of neck narrowing and implant loosening.

## Abbreviations

CT: Computed tomography; FE: Finite element; HRA: Hip resurface arthroplasty; ONFH: Osteonecrosis of the femoral head; THA: Total hip arthroplasty.

## Competing interests

The authors declare that they have no competing interests.

## Authors’ contributions

CLT participated in the design of the study, interpretation of the results and draft of the manuscript. PHH participated in the design of the study and helped with the analysis of data. YCC participated in carrying out the study and reviewing references. All authors read and approved the final manuscript.

## Pre-publication history

The pre-publication history for this paper can be accessed here:

http://www.biomedcentral.com/1471-2474/15/262/prepub
